# HIV Screening Rates among Medicaid Enrollees Diagnosed with Other Sexually Transmitted Infections

**DOI:** 10.1371/journal.pone.0161560

**Published:** 2016-08-24

**Authors:** Oluwatoyosi A. Adekeye, Winston E. Abara, Junjun Xu, Joel M. Lee, George Rust, David Satcher

**Affiliations:** 1 Department of Community Health and Preventive Medicine, Satcher Health Leadership Institute, Morehouse School of Medicine, Atlanta, Georgia, United States of America; 2 National Center for Primary Care, Morehouse School of Medicine, Atlanta, Georgia, United States of America; 3 Department of Health Policy and Management, The University of Georgia, Athens, Georgia, United States of America; Universidade Estadual de Maringa, BRAZIL

## Abstract

**Introduction:**

Approximately 20 million new sexually transmitted infections (STIs) are diagnosed yearly in the United States costing the healthcare system an estimated $16 billion in direct medical expenses. The presence of other STIs increases the risk of HIV transmission. The Centers for Disease Control and Prevention (CDC) has long recommended routine HIV screening for individuals with a diagnosed STI. Unfortunately, HIV screening prevalence among STI diagnosed patients are still sub-optimal in many healthcare settings.

**Objective:**

To determine the proportion of STI-diagnosed persons in the Medicaid population who are screened for HIV, examine correlates of HIV screening, and to suggest critical intervention points to increase HIV screening in this population.

**Methods:**

A retrospective database analysis was conducted to examine the prevalence and correlates of HIV screening among participants. Participant eligibility was restricted to Medicaid enrollees in 29 states with a primary STI diagnosis (chlamydia, gonorrhea, and syphilis) or pelvic inflammatory disease claim in 2009. HIV-positive persons were excluded from the study. Frequencies and descriptive statistics were conducted to characterize the sample in general and by STI diagnosis. Univariate and multivariate logistic regression were performed to estimate unadjusted odds ratios and adjusted odds ratio respectively and the 95% confidence intervals. Multivariate logistic regression models that included the independent variables (race, STI diagnosis, and healthcare setting) and covariates (gender, residential status, age, and state) were analyzed to examine independent associations with HIV screening.

**Results:**

About 43% of all STI-diagnosed study participants were screened for HIV. STI-diagnosed persons that were between 20–24 years, female, residing in a large metropolitan area and with a syphilis diagnosis were more likely to be screened for HIV. Participants who received their STI diagnosis in the emergency department were less likely to be screened for HIV than those diagnosed in a physician’s office.

**Conclusion:**

This study showed that HIV screening prevalence among persons diagnosed with an STI are lower than expected based on the CDC’s recommendations. These suboptimal HIV screening prevalence present “missed opportunities” for HIV screening in at-risk populations. Measures and incentives to increase HIV screening among all STI-diagnosed persons are vital to the timely identification of HIV infection, linkage to HIV care, and mitigating further HIV transmission.

## Introduction

Sexually transmitted infections (STIs) increase the risk of HIV transmission [1, 2]. Persons who are infected with STIs are two-five times more likely than uninfected individuals to acquire HIV through condomless sexual contact [3]. For this reason, the Centers for Disease Control and Prevention (CDC) has long recommended routine HIV screening for individuals with a diagnosed STI [3]. Approximately 20 million new STIs are diagnosed every year in the United States, and this costs the U.S. healthcare system an estimated $16 billion in direct medical expenses [4, 5]. Of this estimated expenditure, nearly 80% ($12.6 billion) is spent on HIV-related costs alone [4]. Medical encounters at the time of STI diagnosis and treatment represent critical opportunities for HIV screening in patients who are high risk, because of their sexual behaviors, and because STIs increase the risk of HIV transmission via genital ulceration, increased HIV viral load in semen, enhanced HIV replication, and altered immune responses [2, 3, 6].

While the CDC recommends universal “opt-out” screening for HIV [7], screening at the time of specific medical indications is an important public health strategy. More than half of U.S. adults between 18 and 55 years have never been tested for HIV [8]. Screening can identify patients infected with HIV, and it facilitates the prompt initiation of antiretroviral therapy (ART), which inhibits progression to AIDS and prevents transmission of the virus. HIV-infected persons are more likely to adopt safer sexual behaviors if they are aware of their HIV status [9, 10]. HIV screening at the time of STI diagnosis is a teachable moment for patients, providing sexual risk reduction counseling to at-risk persons, safe sex resources like condoms or pre-exposure prophylaxis, as well as informing them of their HIV status.

Despite the recommended HIV screening guidelines by the CDC [3], and the merits of HIV screening among STI-diagnosed persons, there is a paucity of recent research examining HIV screening prevalence among STI patients. Evidence shows that there is a failure to adhere to these recommendations in many healthcare settings (STI clinics, emergency departments [ED], and physician outpatient clinics) [11–13]. Therefore, the objective of this study was to determine the prevalence of STI-diagnosed persons in the Medicaid population who are screened for HIV, examine correlates of HIV screening, and to suggest critical intervention points and strategies to increase HIV screening in this population.

Medicaid enrollees represent a sub-population made up predominantly of persons of low socio-economic status with disproportionate representation of racial/ethnic minorities–demographic groups that are disparately burdened by HIV and other STIs [14]. Medicaid provides insurance coverage that eliminates cost barriers to screening. A previous study of Medicaid enrollees with a non-blood-borne STIs (gonorrhea and chlamydia) showed that only 15% were screened for HIV [14]. This low proportion represents a missed opportunity not only at the individual patient level but also in the use of Medicaid claims data for STI/HIV public health surveillance and population-based quality improvement or disease management. At a time when many states are expanding Medicaid and the prevention provisions of the Patient Protection and Affordable Care Act (ACA) have removed cost barriers to HIV screening among many private insurance plans, this study has implications for both publicly and privately insured populations.

## Methods

### Study design, inclusion criteria, and variables

A retrospective database analysis was conducted to identify and analyze HIV screening prevalence among Medicaid enrollees with a primary STI diagnosis (chlamydia, gonorrhea, and syphilis) or pelvic inflammatory disease (PID) claim. PID was included in the analysis because most PID cases are caused by untreated STIs, and some healthcare providers present this claim when a complicated STI is identified. The study population was drawn from a convenience sample of available Medicaid claims data from 29 states (Alabama, Arizona, Arkansas, California, Colorado, Connecticut, Florida, Georgia, Illinois, Indiana, Louisiana, Maryland, Massachusetts, Michigan, Mississippi, Missouri, New Jersey, New Mexico, New York, North Carolina, Ohio, Oklahoma, Pennsylvania, South Carolina, Tennessee, Texas, Virginia, Washington, and Washington, D.C.) between January 1, 2009 and December 31, 2009. Persons from these states make up 90% of all people enrolled in Medicaid and 80% of all black and Hispanic Medicaid enrollees in the entire U.S. Eligibility criteria for the study required participants to: (1) be enrolled in Medicaid throughout 2009 (1/1/2009-12/31/2009), (2) be between 15 and 49 years of age (3) receive a Medicaid claims diagnosis for at least one STI (chlamydia, gonorrhea, and syphilis) or PID, and (4) receive this diagnosis in a physician’s office or the emergency department. STI-diagnosed study participants that were known to be HIV-positive were excluded from the study analysis. Institutional Review Board approval for this study was obtained from the Morehouse School of Medicine, and all patient records/information were anonymized and de-identified prior to analysis.

Using the *International Classification of Diseases*, *Ninth Revision (ICD-9)*, *Clinical Modification* diagnosis codes, individuals who had claims for an STI diagnosis or PID were extracted and evaluated to determine whether they were screened for HIV. HIV screening was defined as having an HIV test performed within 60 days of the primary STI or PID diagnosis. The study’s independent variables were STI diagnosis (gonorrhea, chlamydia, syphilis, and PID), race (white, black, Hispanic, and other [Asian, Native American or Pacific Islander, multiple races or unknown]), and healthcare setting (physician’s office and ED). Participants who identified as Asian, American Indian, Alaska Native, Pacific Islander, multiple races or unknown were categorized as other because of their small sample size.

Age (15–19, 20–24, 25–29, 30–39, and 40–49 years), gender (male and female), residential status (large metropolitan [an area with at least one urbanized area of 50,000 or more inhabitants] [15], small metropolitan [an area with at least one urbanized area with at least 10,000 but less than 50,000 population] [15], and rural [includes areas not included within an urban area]) [16], and states were included as covariates because of their role as conceptual confounders in HIV screening [17]. Participating states were included as covariates because of the varying state eligibility criteria for Medicaid enrollment. Determination of residential status was made by merging each enrollee’s county of residence data from their personal summary file with county-level data from the Area Resource File (ARF) [18]. The ARF is a publicly available health data file that includes environmental and geographical descriptors from which information can be used to characterize a geographic area as large metropolitan, small metropolitan, or rural. The reference group for the independent variables, race, STI diagnosis, and healthcare setting, were white, PID and physician’s office respectively. The reference groups for the covariates were 40–49 years (age), male (gender), rural (residential status), and Georgia (state). The outcome variable was HIV screening within 60 days of the STI diagnosis (yes or no). Because a 60-day window was used to determine HIV screening post-STI diagnosis, STI diagnoses made in the first and last 60 days of the calendar year were excluded to ensure that there was a 60-day window for participants to get screened for HIV.

### Analysis

Frequencies and descriptive statistics were conducted to characterize the sample in general and by STI diagnosis. Univariate and multivariate logistic regression were performed to estimate unadjusted odds ratios (ORs) and adjusted odds ratio (AOR) respectively and their associated 95% confidence intervals (CI). Multivariate logistic regression models that included the independent variables (race, STI diagnosis, and healthcare setting) and covariates (gender, residential status, age, and state) were entered using the entry method to examine their independent associations with HIV screening. A two-tailed level of statistical significance was set at 0.05, and all analyses were conducted using SAS version 9.3 (SAS Institute, Cary, NC) [19].

## Results

Table 1 describes the socio-demographic and STI characteristics of the study participants. The study sample size was made up of 26,672 unique participants. The mean age of respondents was 23.1 years, and most of the study respondents were between 15–19 years (45%), black (62%), female (78%), and resided in a large metropolitan area (58%). Chlamydia was the most diagnosed STI (74%) while gonorrhea (19%), syphilis (5%), and PID (2%) were not as frequently diagnosed. The majority of STI diagnoses were made in the physician’s office (88%). The proportion of chlamydia and gonorrhea cases were highest among participants between 15–19 years while the proportion of syphilis and PID cases were highest among participants between 40–49 years, and 25–29 years respectively. All STIs (chlamydia, gonorrhea, syphilis) and PID were more frequently diagnosed among blacks, females, participants who resided in large metropolitan areas, and in a physician’s office. Patients with a diagnosis of syphilis (roughly 53%) were more frequently screened for HIV compared to patients with a diagnosis of any of the other STIs (chlamydia, gonorrhea, syphilis).

**Table 1:** Demographic Characteristics of STI-diagnosed Persons (15–49 years) Enrolled in Medicaid (n = 26,672) by specific STI: Medicaid Claims Data, United States, 2009.

Characteristic	Chlamydia	Gonorrhea	Syphilis	PID^a^	Total
	n (%)	n (%)	n (%)	n (%)	N
**Overall**	19760 (74)	5165 (19)	1310 (5)	437 (2)	26672
**Age**					
40–49	932 (4.7)	178 (3.5)	387 (29.5)	46 (10.5)	1543
30–39	2160 (10.9)	577 (11.2)	282 (21.5)	102 (23.3)	3121
25–29	2692 (13.6)	738 (14.3)	171 (13.1)	108 (24.7)	3709
20–24	4724 (23.9)	1318 (25.5)	174 (13.3)	99 (22.7)	6315
15–19	9252 (46.8)	2354 (45.6)	296 (22.6)	82 (18.8)	11984
**Race**					
White	3974 (20.1)	747 (14.5)	265 (20.2)	141 (32.3)	5127
Black	12017 (60.8)	3751 (72.6)	701 (53.5)	189 (43.3)	16658
Hispanic	1715 (8.7)	253(4.9)	126 (9.6)	67 (15.3)	2161
Other	2054 (10.4)	414 (8.0)	218 (16.6)	40 (9.2)	2726
**Gender**					
Male	4331 (21.9)	995 (19.3)	468 (35.7)	*	5796
Female	15429 (78.1)	4170 (80.7)	842 (64.3)	435 (99.5)	20876
**Residential status**					
Rural	3011 (15.2)	615 (11.9)	114 (8.7)	79 (18.1)	3819
Large metropolitan area	11131 (56.3)	3060 (59.3)	908 (69.3)	272 (62.2)	15371
Small metropolitan area	5618 (28.4)	1490 (28.9)	288 (22.0)	86 (19.7)	7482
**Practice setting (where first STI was diagnosed)**					
Physician’s office	17361 (87.9)	4444 (86.0)	1176 (89.8)	362 (82.8)	23343
ED^b^ visit	2399 (12.1)	721 (14.0)	134 (10.2)	75 (17.2)	3329
**HIV screening (≤60 days)**					
Yes	8481 (42.9)	2094 (40.5)	697 (53.2)	186 (42.6)	11458
No	11279 (57.1)	3071 (59.5)	613 (46.8)	251 (57.4)	15214

^a^ Pelvic inflammatory disease

^b^ Emergency Department

* Missing responses in this cell

The results of univariate and multivariable logistic regressions are presented in Table 2. Overall, about 43% of participants with a diagnosis of STI were screened for HIV. Several factors were associated with HIV screening in the univariate model. The significant predictors for a higher likelihood of HIV screening were participants who were female, (OR = 1.27, 95% CI = 1.20–1.35) or who received a diagnosis of syphilis, (OR = 1.53, 95% CI = 1.23–1.91).

**Table 2:** Univariate (Unadjusted) and Multivariable (Adjusted) Logistic Regression Model Predicting HIV Screening Prevalence Among STI-diagnosed Persons (15–49 years): Medicaid Claims Data, United States, 2009.

Variable	% HIV screened	Unadjusted OR (95% CI)	P Value	Adjusted OR^a^ (95% CI)	P Value
**Overall**	42.9 (42.4–43.6)	N/A	N/A	N/A	N/A
**Age**					
40–49	41.5 (39.0–43.9)	1 (N/A)	N/A	1 (N/A)	N/A
30–39	42.0 (40.3–43.8)	1.02 (0.90–1.16)	0.7172	1.12 (0.98–1.28)	0.0879
25–29	42.2 (40.7–43.8)	1.03 (0.92–1.16)	0.6086	1.14 (1.00–1.30)	0.0560
20–24	42.9 (41.6–44.1)	1.06 (0.95–1.18)	0.3307	1.14 (1.01–1.29)	0.0417
15–19	43.7 (42.8–44.6)	1.09 (0.98–1.22)	0.1034	1.12 (1.00–1.26)	0.0614
**Race**					
White	44.1 (42.7–45.4)	1 (N/A)	N/A	1 (N/A)	N/A
Black	42.5 (41.8–43.3)	0.94 (0.88–1.00)	0.0513	0.95 (0.89–1.02)	0.1585
Hispanic	40.6 (38.5–42.7)	0.87 (0.78–0.96)	0.0062	1.12 (0.99–1.26)	0.0650
Other	45.5 (43.6–47.3)	1.06 (0.96–1.16)	0.2380	1.09 (0.98–1.21)	0.1111
**Gender**					
Male	38.5 (37.2–39.7)	1 (N/A)	N/A	1 (N/A)	N/A
Female	44.2 (43.5–44.9)	1.27 (1.20–1.35)	<0.0001	1.16 (1.09–1.24)	<0.0001
**Residential status**					
Rural	53.2 (51.6–54.8)	1 (N/A)	N/A	1 (N/A)	N/A
Large metropolitan area	41.1 (40.4–41.9)	0.62 (0.57–0.66)	<0.0001	1.24 (1.13–1.36)	<0.0001
Small metropolitan area	41.5 (40.4–42.6)	0.62 (0.58–0.67)	<0.0001	0.95 (0.86–1.04)	0.2645
**STI diagnosis**					
PID	42.6 (37.9–47.2)	1 (N/A)	N/A	1 (N/A)	N/A
Chlamydia	42.9 (42.2–43.6)	1.02 (0.84–1.23)	0.8814	0.92 (0.75–1.13)	0.4398
Gonorrhea	40.5 (39.2–41.9)	0.92 (0.76–1.12)	0.4091	0.92 (0.74–1.13)	0.4133
Syphilis	53.2 (50.5–55.9)	1.53 (1.23–1.91)	0.0001	1.59 (1.27–2.01)	<0.0001
**Practice setting (where STI was initially diagnosed)**					
Physician’s office	45.7 (45.0–46.3)	1 (N/A)	N/A	1 (N/A)	N/A
ED visit	24.0 (22.5–25.5)	0.38 (0.35–0.41)	<0.0001	0.41 (0.38–0.45)	<0.0001

^a^ adjusted for state; OR = odds ratio; AOR = Adjusted odds ratio; 95% CI = 95% confidence interval

Conversely, Hispanic participants (OR = 0.87, 95% CI = 0.78–0.96), participants residing in a large metropolitan area (OR = 0.62, 95% CI = 0.57–0.66), participants residing in a small metropolitan area (OR = 0.62, 95% CI = 0.58–0.67), and participants who received an STI diagnosis in the ED (OR = 0.38, 95% CI = 0.35–0.41) were less likely to be screened for HIV.

In the multivariable analysis, differences in HIV screening remained significant by STI diagnosis and healthcare setting but not by race. There were no significant differences in HIV screening between white participants and black (AOR = 0.95, 95% CI 0.89–1.02), Hispanic (AOR = 1.12, 95% CI 0.99–1.26) and other participants (AOR = 1.09, 95% CI 0.98–1.21). Participants that were between 20–24 years (AOR = 1.14, 95% CI 1.01–1.29), female (AOR = 1.16, 95% CI 1.09–1.24), residing in large metropolitan areas (AOR = 1.24, 95%CI 1.13–1.36), and a diagnosis of syphilis (AOR = 1.59, 95% CI 1.27–2.01) were significantly more likely to be screened for HIV. On the other hand, receiving an STI diagnosis in the ED was associated with a lower likelihood of HIV screening (AOR = 0.41, 95% CI 0.38–0.45).

## Discussion

The results of this study demonstrate that about 43% of STI-diagnosed patients were screened for HIV; far less than the expected proportion of STI-diagnosed persons screened for HIV considering the current CDC guidelines [3]. The gap between usual-care and guideline-appropriate screening behaviors affects a large number of people at increased risk of contracting HIV. An estimated 1.4 million chlamydial cases, 350,000 gonococcal cases, and about 63,000 cases of syphilis were reported in the US in 2014 [20]. Almost 50,000 new HIV cases are diagnosed annually in the US [21]. Research has indicated that only around 7% of Medicaid-enrolled patients are screened for both syphilis and HIV [14], while 11% and 10% of Medicaid-enrolled patients diagnosed with chlamydia and gonorrhea respectively are screened for HIV [14].

Despite the established relationship between syphilis and HIV infections, only about 53% of the participants diagnosed with syphilis in this study were screened for HIV. Furthermore, barely 41% of the participants diagnosed with gonorrhea, and 43% of those diagnosed with chlamydia, were screened for HIV. Overall, study participants diagnosed with syphilis were the most likely to be screened for HIV, a determination consistent with other documented findings, which showed that persons with a syphilis diagnosis reported the highest HIV screening prevalence of all STI-diagnosed persons [9, 22]. This trend may be explained by clinical workflows, which include HIV screening as an additional lab request for a patient who is already receiving a blood-draw for syphilis testing. HIV-screening after a diagnosis of gonorrhea or chlamydia, in contrast, requires the extra clinician effort to send the patient to the lab for a specific blood draw (both for syphilis and HIV-screening). The failure to screen for HIV among at-risk persons represents important missed opportunities to identify persons infected with HIV, make them aware of their HIV status, and promptly connect them with HIV care. HIV-infected persons unaware of their HIV status are 3.5 times more likely to transmit HIV than persons who are aware their status [23].

System-level interventions, including approaches utilized in offering HIV tests, might be beneficial in increasing HIV-screening, at least, in persons diagnosed with syphilis. These strategies could include the “opt-out approach”- where patients are informed that they will be tested for HIV, except they decline, or standing orders that require HIV screening in all persons that are diagnosed with an STI [3, 24, 25].

Several studies have examined HIV screening prevalence in STI-diagnosed persons with varying results. A 2005 survey of 80 commercial health plans showed an overall HIV screening prevalence of almost 20% [22]; while a 2006–2007 survey of six health insurance plans indicated an overall HIV screening prevalence of nearly 33% [9]. In 2009–2010, another study using Sexually Transmitted Diseases (STD) surveillance network data reported a HIV screening prevalence of 51% [26], which was similar to a 2014 survey of Veterans Health Administration (VHA) administrative data which showed a 45% HIV screening prevalence [10]. Our study showed a rather sizable improvement from the 15% HIV screening prevalence recorded in a similar study that utilized 1998 Medicaid claims data from four states [14]. This likely reflects improvement in clinician practice behaviors specific to high-risk patients, or represent a halo effect from the CDC universal (“opt-out”) HIV-screening recommendation made in 2006 [7].

Interventions that focus on healthcare providers are critical to increasing HIV screening prevalence among patients with a diagnosed STI. In particular, educational approaches geared toward improving provider awareness on the association between STIs and HIV is needed. Likewise, other educational campaigns that could increase the prevalence at which providers comply with the CDC recommended guidelines on HIV screening among patients with any STI in all healthcare settings is needed to address the disparate screening prevalence by STI. At the population level, viewing Medicaid as a public health surveillance tool that could inform the implementation of improved practices and policies, in addition to being a payer of insurance claims may be more impactful than the traditional clinician feedback and education interventions [27].

This study did not detect differences in HIV screening prevalence by race, a finding consistent with other cohort studies that have utilized Medicaid data [28, 29]. The failure to detect racial differences may be due to the relative socioeconomic homogeneity of low-income Medicaid populations. Furthermore, all study participants presumably had the same level of health insurance coverage and access to health care during the study period. In any case, Medicaid appears to be an equalizing force with regard to health disparities [28].

Data from this study also identified differential screening prevalence by practice settings. Persons diagnosed with an STI in a physician’s office were almost twice as likely to be screened for HIV compared to those who received their diagnosis in the ED. This evidence is supported by another study conducted in 2011, which documented a lower HIV screening prevalence among STI-diagnosed persons in the ED [9]. This finding could be a result of the increased familiarity and relationship that physicians sometimes develop with their patients in an office setting, which may, in turn, influence HIV screening prevalence. The prospect of additional demand on ED providers’ time (pre-test counseling, HIV screening, and post-test HIV counseling), especially in a time-pressured ED environment, coupled with key barriers to HIV screening in the ED, such as the difficulty with follow-up [30] may discourage HIV screening in this healthcare setting. Moreover, insurers might be unwilling to pay for HIV tests in the ED if they are considered unrelated to the primary complaint. Many ED providers might also be averse to HIV screening among STI-diagnosed persons because they are trained to focus on acute illnesses or life-threatening injuries. Perhaps, these barriers could be mitigated by integrating HIV screening and case management within the ED, or case referrals from EDs to settings primarily focused on HIV screening and case management. Programs that facilitate rapid HIV screening such as expanding the availability of rapid HIV testing in the ED may also be beneficial. Institutional changes, such as electronic health record prompts, ED provider education regarding HIV screening, and the utilization of appropriate approaches to offering HIV tests to patients are also useful [24, 31]. Finally, prevention provisions of the Affordable Care Act that mitigate HIV screening test costs to patients could facilitate HIV screening prevalence across healthcare settings [32].

## Limitations

Limitations of this study are those inherent in Medicaid claims data research. The findings are primarily generalizable to the Medicaid-enrolled population at the time of the study, and to the Medicaid programs that pay for their care. Because of the categorical as well as needs-based requirements for Medicaid participation in the study year (2009), the population sampled was disproportionately younger, minority, and female, compared to the general U.S. population. Data available were for events that were paid for in Medicaid claims and, therefore, could not include STI-diagnosed patients who might have screened for HIV elsewhere. Besides, patients who were offered an HIV test but declined could not be accounted for in this study. Finally, since secondary data were utilized, this study was unable to account for the factors responsible for the variance in the outcomes observed such as providers and patients’ HIV risk perceptions and providers’ subjective risk assessment [33, 34]. Despite these limitations, this study has its strengths. Though only Medicaid claims data from 29 states were used, these states are population-dense, and they represent all claims on 80% of all U.S. Medicaid enrollees and an even greater proportion of minority Medicaid enrollees in the nation. Another strength of this study is that it reflects real-world screening behaviors without response bias, in contrast to self-reported behaviors or studies in which clinicians or patients know they are being observed.

## Conclusion

HIV screening among STI-diagnosed persons is an underutilized public health strategy. This study showed that HIV screening prevalence among persons diagnosed with an STI are lower than expected based on the CDC’s recommendations. These suboptimal screening prevalence present “missed opportunities” for HIV screening in at-risk populations. Measures and incentives to increase HIV screening among all STI-diagnosed persons are vital to the timely identification of HIV infection, linkage to HIV care, and mitigating further HIV transmission.

In the broader U.S. population, this study adds to the weight of evidence supporting the urgent need for the development and implementation of standard quality improvement protocols that will support adherence to the CDC recommendation for routine HIV screening. These include provider HIV/STI education and awareness, integrated HIV/STI services and case management, collaborative partnerships with HIV/STI public health departments, as well as ensuring electronic health record prompts for HIV screening. Other strategies include training and encouraging healthcare providers to engage routinely in discussions with their STI-diagnosed patients about HIV screening [35], and the wider adoption of rapid, non-invasive HIV screening tests at the point of care [36], Results of this study also demonstrate the specific ability of Medicaid claims as well as other payer claims data to provide on-going surveillance of STIs that can be used by state Medicaid programs and public health departments to significantly improve HIV-screening behaviors at the population level. This requires a greater level of collaboration and integration between traditional public health units and state Medicaid programs than currently exists in many states. Lastly, concerted efforts are needed to increase HIV screening prevalence in all health care settings among this at-risk population.
